# Economic analysis of the adoption of capsule endoscopy within the British NHS

**DOI:** 10.1093/intqhc/mzaa039

**Published:** 2020-05-12

**Authors:** Alan Lobo, Rafael Torrejon Torres, Mark McAlindon, Simon Panter, Catherine Leonard, Nancy van Lent, Rhodri Saunders

**Affiliations:** 1 Academic Dept of Gastroenterology, Sheffield Teaching Hospitals NHS Foundation Trust, Sheffield, UK; 2 Coreva Scientific, Koenigswinter, Germany; 3 Department of Gastroenterology, South Tyneside NHS Foundation Trust, South Shields, UK; 4 Medtronic UK, Watford, UK

**Keywords:** economic evaluation (cost-effectiveness), general methodology, simulation and modelling care pathways/disease management, appropriate health care, digestive diseases, disease categories

## Abstract

**Objective:**

Identification of a cost-effective treatment strategy is an unmet need in Crohn’s disease (CD). Here we consider the patient outcomes and cost impact of pan-intestinal video capsule endoscopy (PVCE) in the English National Health Service (NHS).

**Design:**

An analysis of a protocolized CD care pathway, informed by guidelines and expert consensus, was performed in Microsoft Excel. Population, efficacy and safety data of treatments and monitoring modalities were identified using a structured PubMed review with English data prioritized. Costs were taken from the NHS and Payer Provided Services (PSS) 2016–17 tariffs for England and otherwise literature. Analysis was via a discrete-individual simulation with discounting at 3.5% per annum.

**Setting:**

NHS provider and PSS perspective

**Participants:**

4000 simulated CD patients

**Interventions:**

PVCE versus colonoscopy ± magnetic resonance enterography (MRE)

**Main outcome measures:**

Costs in 2017 GBP and quality-adjusted life years (QALY)

**Results:**

The mean, total 20-year cost per patient was £42 266 with colonoscopy ± MRE and £38 043 with PVCE. PVCE incurred higher costs during the first 2 years due to higher treatment uptake. From year 3 onwards, costs were reduced due to fewer surgeries. Patients accrued 10.67 QALY with colonoscopy ± MRE and 10.96 with PVCE. PVCE dominated (less cost and higher QALY) colonoscopy ± MRE and was likely (>74%) to be considered cost-effective by the NHS. Results were similar if a lifetime time horizon was used.

**Conclusions:**

PVCE is likely to be a cost-effective alternative to colonoscopy ± MRE for CD surveillance. Switching to PVCE resulted in lower treatment costs and gave patients better quality of life.

## Background

Crohn’s disease is a chronic condition characterized by inflammatory activity within the gastrointestinal tract, affecting both the colon and small bowel. It is estimated that at least 114 000 people suffer from Crohn’s disease in the UK, associated with a mean annual cost to the National Health Service (NHS) of £6156 per patient [1, 2]. In a UK database study, 47% of Crohn’s patients had required surgical intervention as a treatment for their disease [3]. This suggests that optimal management of Crohn’s disease remains a challenge.

NICE guidelines emphasize induction and maintenance of remission based on symptom relief or improvement achieved through administration of anti-inflammatory or immunomodulating medication [2]. However, achieving mucosal healing or endoscopic remission is now recognized as important and may be associated with improved long-term outcomes [4–6]. The effect of different management approaches in the CALM study showed that a tight, symptom- and biomarker-driven disease management led to superior outcomes compared to symptomatic assessment only [7].

To assess endoscopic remission, visualization of the gastrointestinal tract is necessary. This is usually achieved using colonoscopy, an invasive procedure generally considered unpleasant by patients [8]. To reduce the need for colonoscopies, biomarker and clinical assessment are recommended screening procedures prior to referral for colonoscopy [2, 9, 10]. Faecal calprotectin is sensitive for Crohn’s disease inflammation and is considered an indicator of disease activity [9, 11, 12]. Its introduction in England substantially reduced patient referrals for colonoscopies [13].

Colonoscopy allows assessment of the colon and terminal ileum. One in 10 patients, however, has an exclusive or additional disease activity in the small bowel [3]. Additional imaging such as magnetic resonance enterography (MRE) may therefore be required, with the disadvantage of lacking direct visualization of the mucosa [14]. Pan-intestinal video capsule endoscopy (PVCE), a noninvasive, direct-visualization procedure requiring no sedation, lowers the burden on patients [15]. Commonly reported adverse events are capsule retention in 0—13.6% of patients with established inflammatory bowel disease [16]. Using a patency capsule to detect strictures, retention rates decrease to 4% [16]. Recently, a PVCE system designed specifically to identify signs of Crohn’s disease activity (PillCam™ Crohn’s system Medtronic Inc) was launched, and results from early adopters showed a sensitivity comparable to the gold standard colonoscopy [17].

This study aims to assess the costs and consequences resulting from a change in current disease monitoring practice, specifically if colonoscopy and additional imaging were to be replaced (in eligible patients) by PVCE. In this way, the aim is to help inform key questions identified by both physicians and patients, improving ‘cost-effectiveness in IBD management’ and ‘monitoring disease activity’ [18].

## Methods

### Representation of a common national care pathway

In the UK, patient care is split between primary and secondary providers. Patients with a diagnosis of Crohn’s disease have, in most cases, a secondary care gastroenterologist guiding treatment. In this analysis we model the impact of changing the common monitoring practices used by these gastroenterologists.

**Figure 1:**
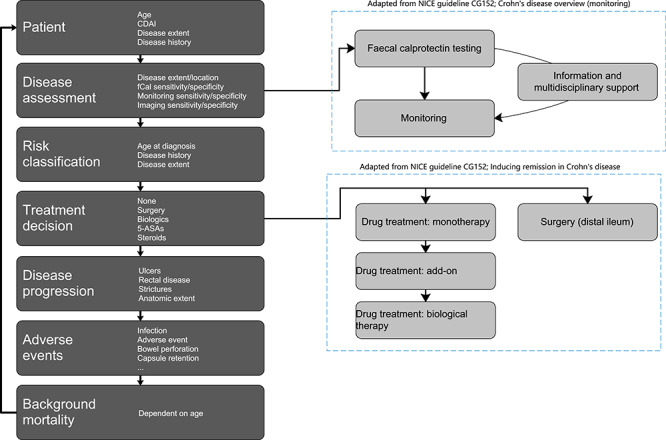
Care pathway. A schematic of a patient’s progression through the model. In the ‘Patient’ section, the patient’s initial characteristics and disease state are first generated to be within the confidence interval of the mean value of the UK Crohn’s population. In subsequent years of the model, the ‘Patient’ section of the model is used to update the patient’s characteristics (e.g. increasing age). It is then determined if the patient is scheduled for clinical review or has symptomatology necessitating a clinical review. If a clinical review is to be undertaken, the patient goes to the ‘Disease assessment’ section and if not goes directly to ‘Disease progression’. In the ‘Disease Assessment’ section, the patient has a clinical review by a physician, which includes a faecal calprotectin test and monitoring if deemed necessary. Based upon the symptomatology and patient history, a low-, moderate- or high-risk classification is set in the ‘Risk classification’ section. Next, a ‘Treatment decision’ is made. If the patient is diagnosed with active Crohn’s disease, or is currently receiving maintenance treatment, a decision on continuation or assigning a new treatment is made. This is a step-up to the next treatment line if the patient is already on therapy. If the patient currently has no active treatment, then a new, initial treatment is assigned dependent on the patient’s risk classification. Surgery can also be prescribed at this stage; this occurs when the disease extent is too excessive and/or the current lines of therapy have failed. At the next stage, ‘Disease progression’ (remission, no change or worsening) is simulated in all patients. The likelihood of the different types of progression is informed by current treatment and patient risk factors. Any ‘adverse events’ that have affected the patient over the model cycle are simulated in the next section of the model. The model cycle completes with the assessment of mortality due to non-Crohn’s causes in the ‘Background mortality’ section. The probability of death per year of life is assigned from English life tables.

As an initial step to understand current care provision, the NICE guidelines were reviewed and a panel of UK experts consulted. The Crohn’s disease care pathway described below was protocolized following meeting and survey of the panel of seven physicians based in England. Their responses were aggregated, and either the median or modal answer was incorporated into the NICE clinical pathway (Fig. 1). In the analysed pathway, the patient has regular scheduled appointments with their gastroenterologist. The interval between appointments is dependent on the patients’ past disease activity (those with severe disease are seen more frequently). Regular scheduled appointments can be moved forward if symptomatology flares.

**Table 1:** Efficacy and safety data

	fCal test	PVCE^a^	Ileocolonoscopy	MRE
**Sensitivity** ^b^	78.8 [43]	93% [44] SB 100% [35]	90% [45] SB -	81% [35]
**Specificity** ^b^	97.2 [43]	84% [44] SB 91% [35]	100% [45] SB -	86% [35]
**Subsequent hospitalization**	-	-	1.63% [46]	0
**Bowel obstruction**	*-*	^c^	0.08% [47]	0
**Gastrointestinal bleeding**	-	-	0.42% [48]	0
**Infection**	-	-	4% [49]	0
**Capsule retention**				
**- With PC**	-	2.1% [16]	-	-
**- Without PC**	-	8.2% [16]	-	-
**Complete procedures**	100%	88.7% [23]	86.9% [24]	100%

MRE, magnetic resonance enterography; PC, patency capsule; SB, small bowel; SBC, small bowel and colon video capsule endoscopy. Together colonoscopy and MRE form what is here considered common monitoring practice (CMP).

a Not all data are specific to PVCE; some data come from studies of generic VCE devices.

b Sensitivity and specificity are defined in individual studies but in general reflect accurate diagnosis relative to the gold standard used in the referenced study.

c Capsule retention can result in bowel obstruction. To account for this in the model, 5% of capsule retention events require surgical removal.

**Table 2:** Population characteristics

Characteristic	Mean	Source
**Age**	**42**	[40]
**Age at diagnosis**	**30**	[3]
**Gender, % male**	**42**	[3]
**Weight**	**75.83**	[41]
**CDAI score**	**220**	**Mild threshold**
**Ileal (L1), %**	**39.25** ^a^	[3]
**Colonic (L2), %**	**23.25** ^a^	[3]
**Ileocolonic (L3), %**	**25.25** ^a^	[3]
**Upper GI (L4), %**	**14** ^a^	[3]
**Superficial ulcers, %**	**30**	Illinois Gastroenterology Group Project Sonar Database
**Deep ulcers, %**	**13**	[40]
**Severe rectal disease, %**	**9**	[40]
**Stricture, %**	**22.87**	Illinois Gastroenterology Group Project Sonar Database
**Anatomic involvement, %**	**34**	[3]
**Previous GI haemorrhage, %**	**5**	Assumption
**Previous surgery**	**47**	[3]

a Only highest disease location considered. Other and unknown distributed evenly between all four locations

CDAI, Crohn’s disease activity Index; GI, gastrointestinal

At each appointment, the gastroenterologist assesses the patient’s disease in up to three ways:

- A: Through discussion with the patient regarding symptomatology and health status. If there is suspicion of active disease, move to B.
- B: Faecal calprotectin checked against established cut-offs [11, 22]. If negative, conclude appointment. If positive, consider C unless the patient was recently monitored and/or is receiving active treatment.
- C: Monitoring with colonoscopy ± MRE—the predominant imaging methodology within the UK.

With these information, the physician determines disease status (remission or active), severity (mild, moderate, severe), and requirement for treatment (pharmaceuticals, biologics, surgery, none). When treatment is prescribed, a step-up approach is taken, based on patient risk, moving through the following lines:

- 1: Corticosteroids (low-risk patients, ≤ 1 risk factor, start treatment here)
- 2: Corticosteroids + azathioprine (moderate-risk patients, 2 risk factors, start here)
- 3: Infliximab + azathioprine (high-risk patients, > 2 risk factor, start here)
- 4: Adalimumab
- 5: Vedolizumab

where risk factors are age < 30 years at initial diagnosis, extensive anatomic involvement, prior resection, perianal and/or severe rectal disease, deep ulcers, and stricturing and/or penetrating behaviour.

### Decision analytic model development

Crohn’s disease represents a challenge with non-uniform symptomatology and treatment decisions made based on combinations of evidence specific to each individual patient. As such a discrete-individual simulation (as per Brennan *et al*.) [21] including 4000 patients was taken. Using this approach, the patients’ full disease history, including phases of remission, flares, and adverse events occurring, is available to inform any clinical decisions. The model, developed in Microsoft Excel®, conforms with ISPOR good practice [19], and full details are available in Saunders *et al*. [20]

Using 4000 individual patients in the model, the analysis can be viewed as a proxy for a prospective, observational study. The impact of switching treatment practices can be assessed without exposing any patients to potential harm. Here, the simulation is run twice. In the first instance, patients receive colonoscopy ± MRE when required as the third step in the gastroenterologists’ investigations. In the second instance, the same patient would receive PVCE instead of colonoscopy ± MRE.

As the patient’s care is dictated in part by the disease activity, a model for Crohn’s disease progression and regression was needed. A separate Markov model underlying the discrete-individual simulation was used to estimate progression and regression of Crohn’s disease. The transitions between Markov states are modulated by patient characteristics and current treatments. At every time point in the simulation, it is ‘known’ in the model whether a patient has active disease or symptomatology. Based on the gastroenterologist-collected information and, hence, the determined diagnosis (which may or may not match the actual patient’s health status in the model), patients are grouped into those receiving:

- No active treatment:
  - Endoscopically and clinically disease free (remission)
  - Asymptomatic active disease
  - Symptomatic active disease
  - Symptomatic non-active disease
- Active treatment: maintenance treatment and treatment failure
- Surgery: surgery and post-surgery

### Patient follow-up

Every 3 months the patient health status is updated based on Crohn’s disease progression/regression, and a new Crohn’s Disease Activity Index (CDAI) score is calculated. If the CDAI flares or the patient is scheduled to see the gastroenterologist, the care pathway model is initiated.

### Monitoring adverse events

Adverse events and their incidence from literature review are provided in Table 1. Both colonoscopy and PVCE require an extensive bowel preparation procedure that is for modelling purposes considered to be equal for both procedures. Adverse events from procedural sedation necessary for colonoscopy were not modelled. Capsule retention was modelled using three scenarios:

- The basecase: PVCE is performed only in those patients in whom a prior patency capsule was successfully passed. In these patients the risk of capsule retention during PVCE is 2.1% [16]. If patency capsule failed, the patients switched to colonoscopy ± MRE for the current and all future assessments.
- Scenario 1: As the basecase, the switch to colonoscopy ± MRE is only a one off.
- Scenario 2: No patency capsule is used, and the risk of capsule retention during PVCE is 8.2% [16]. Capsule retention is treated as per the basecase.

Where capsule retention did occur, it was assumed to be treated using endoscopic techniques, but in 5% of cases, surgical resection is required.

### Input data

The model’s patient population and clinical data were taken from peer-reviewed published literature or, if not available, based on expert input. Prioritized during the data collection was data directly from England. Otherwise data were taken from publications on similar, developed health care systems. Key population data used in the model are shown in Table 2.

**Table 3:** Cost data

Item	Costs (GBP)	QALY
**Ileocolonoscopy, per procedure**	654.18^a^	0.0025
**PVCE, per procedure**	800.75^a^^,^^d^	0.0014
**MRE, per procedure**	206.51^a^	
**CTE, per procedure**	120.07^a^	
**Faecal calprotectin test, per procedure**	123.33^a^^,^^e^	
**Clinical assessment of symptoms, per procedure**	108^e^	
**Infliximab, per year**	5278 (NICE TA187)	0.00032
**Infliximab, administration cost**	225	
**Adalimumab, per year**	2860 (NICE TA187)	0.00052
**Adalimumab, administration costs**	0	
**Vedolizumab, per year**	24 350 (NICE TA352)	0.00032
**Vedolizumab, administration costs**	310 (NICE TA352)	
**Corticosteroids, per year**	90 (NICE CG152)	
**Corticosteroids, administration costs**	0	
**Azathioprine, per year**	4 (methotrexate used)	
**Azathioprine, administration costs**	0	
**Inflixmab + azathioprine, per year**	5282	0.00032
**Infliximab + azathioprine, administration costs**	225	
**Remission**	10	0.8 [49]
**Non-active symptomatic**	30	0.61 [49]
**Active symptomatic**	50	0.5 [49]
**Active non-symptomatic**	20	0.8 [49]
**Surgery**	16 583 [1]	0.022 [50]
**Abscess drainage**	2569^a^	
**Fistula repair**	2951^a^	
**Stricturing repair**	2951^a^	
**Capsule retention** ^b^	645^a^	0.0025^c^ [51]
**Bowel perforation**	8797^a^	0.010 [52]

CTE, computed tomography enterography; MRE, magnetic resonance enterography; PVCE, pan-intestinal video capsule endoscopy

a Reference Cost Collection: National Schedule of Reference Costs, 2016–17—NHS trusts and NHS foundation trusts

b 5% of capsule retentions are treated as required for surgery.

c Removal via push-endoscopy.

d Costs include GBP 758.75 for the PVCE procedure and GBP 42 for the patency capsule*.*

e Costs including follow-up attendance with a single gastroenterologist (code 301, NHS England National Prices and Tariffs Workbook 2016/17).

**Table 4:** Results of the model

	Colonoscopy	Basecase: PVCE with patency, permanent conversion after retention	Scenario 1: PVCE with patency, one time conversion after retention	Scenario 2: PVCE no patency, permanent conversion after retention
**Costs**	**£42 266**	**£38 043**	**£37 880**	**£39 772**
**Cost/year**	**£ 2191**	**£1960**	**£1953**	**£ 2055**
**QALE**	**10.67**	**10.96**	**10.96**	**10.84**
**LE**	**19.29**	**19.41**	**19.39**	**19.35**
**Sensitivity**				
**Dominant,%**		**38.25**	**30.55**	**47.7**
**Cost-effective,%**		**35.95**	**37.25**	**30.45**
**Non cost-effective,%**		**23.85**	**30.30**	**19.50**
**Dominated,%**		**1.95**	**1.90**	**2.40**

QALE, quality-adjusted life expectancy; LE, life expectancy; PVCE, pan-intestinal video capsule endoscopy. Model results. The sensitivity analysis is averaging 2000 populations of 50 patients each.

All costs the UK’s NHS and Prescribed Specialised Services (PSS) can expect to pay over 20 years are included and assessed. Costs and quality of life utilities (generally EQ-5D) were discounted at 3.5% per annum after year 1. Costs are in 2016 units of currency. See Table 3 for further details.

### Sensitivity analyses

The robustness of results was explored by varying every input parameter, every cycle. From the individual patient outcomes, bootstrapped populations (random selection with replacement) were created for the analysis of the percentage of cost-saving or cost-effective simulations and the 95% credible interval (CrI) for costs and QoL. Bootstraps were performed 2000 times with populations of 50 patients created. Finally, an analysis over the lifetime horizon, as opposed to 20 years in the basecase, was performed.

The willingness-to-pay threshold for NICE is generally accepted to be an incremental cost-effectiveness ratio (ICER) between £20 000 and £30 000 per quality-adjusted life year (QALY) gained. In this analysis a lower willingness-to-pay threshold of £10 000 per QALY gained is used. This provides a more conservative estimation of cost-effectiveness and is the ICER for consideration for fast track appraisals by NICE, the point at which a product is considered to provide ‘exceptional value for money’.

## Results

Model estimates put the mean annual cost of care per patient receiving standard of care at £2191 (Table 4). The total mean cost per patient over the 20-year time horizon was £42 266. Cost of care with PVCE did not vary substantially from that with colonoscopy, being on average £1960 per patient per year. Mean total costs over 20 years with PVCE were £38 433. The number of endoscopic procedures per patient per year was 0.77 and 0.70 for colonoscopy and PVCE, respectively.

**Figure 2:**
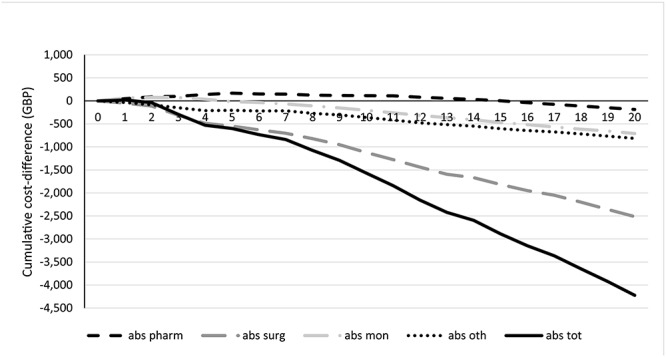
Cumulative absolute difference between standard of care and PVCE. Cumulative, absolute difference between colonoscopy ± MRE and PVCE. Absolute costs are split for monitoring (abs mon), pharmaceutical (abs pharm), surgical (abs surg) and ‘other’ (abs oth). Pharmaceutical and monitoring costs are higher with PVCE than with colonoscopy ± MRE for 15 and 5 years, respectively. Costs attributable to surgical costs, both elective and emergency, are reduced with PVCE. Over the first 2 years, colonoscopy ± MRE is associated with lower total costs (abs tot), while afterwards PVCE is continually less costly.

Over the first year the cumulative costs for PVCE are higher than with colonoscopy (Fig. 2). These cost increases are driven by the higher costs of PVCE compared to colonoscopy and a higher number of patients on active treatment compared to colonoscopy. From 2 years onwards, the cumulative costs for PVCE are lower than colonoscopy. At this point, the increased costs for pharmaceuticals attributable to PVCE become offset by the lower cost for surgeries and other interventions. Over 20 years, the mean per patient cost of surgical interventions is approximately £4200 lower with PVCE than with colonoscopy.

**Figure 3:**
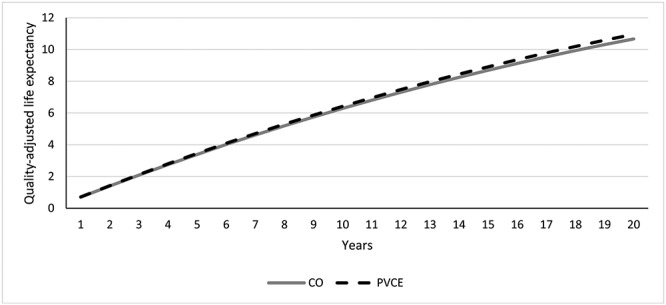
Cumulative QALY. Cumulative, average quality-adjusted life expectancy (QALE), measured in quality-adjusted life years (QALYs), is depicted for the simulated population. Over the first years, the QALE is fairly similar between colonoscopy ± MRE and PVCE. Over the whole time horizon, the QALE improves by 0.31 QALYs with the use of PVCE.

**Figure 4:**
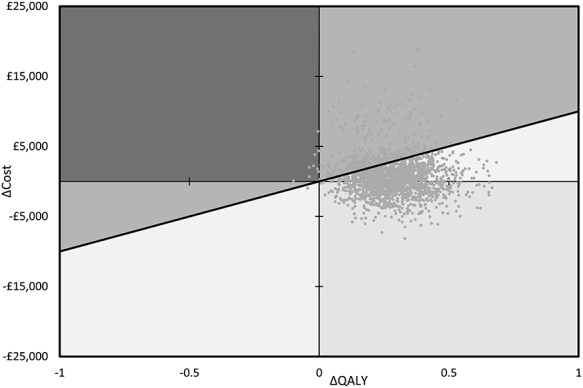
Cost-effectiveness plane. Cost-effectiveness plane for the basecase scenario assuming patency capsule use and permanent switch to colonoscopy upon a capsule retention event. Each point on the graph is the result from a single analysis using random sampling of model parameters. The thick, black, diagonal line depicts the willingness-to-pay threshold which is set to 10 000 GBP per QALY. Each point above the line is a non-cost-effective result, while points below the line are cost-effective.

With colonoscopy 10.67 QALYs were accrued over the 20 years, with PVCE accruing an additional 0.29 (total 10.96) QALYs. Cumulative over the 20-year horizon, the QALY difference is positive for PVCE from year 1 onwards (Fig. 3). The increase in QALYs and the decrease in costs result in PVCE dominating colonoscopy. Overall PVCE was likely to be considered cost-effective in the UK, with 74.2% of bootstrapped patient simulations falling under the willingness-to-pay threshold of £10 000 per QALY gained (Fig. 4).

Scenario 1: The costs for patients in the PVCE arm were reduced compared to the basecase scenario, and the gain in QALYs was increased (Table 4). This suggests that a one off switch to colonoscopy ± MRE is preferable, albeit only slightly, compared to a permanent switch. However, fewer simulations would be considered cost-effective (67.8%).

Scenario 2: Without use of patency capsule, PVCE still led to cost savings and an increase of QALY compared with colonoscopy ± MRE. For both costs and QALYs, the benefits were lower than in the basecase (Table 4). In sensitivity analyses 78.2% of populations were considered as being cost-effective, of which over half of scenarios were dominant.

**Life-long time horizon:** The results were aligned with the basecase as PVCE reduced care costs and increased quality of life. The average costs of care per patient were GBP 52 212 using colonoscopy ± MRE and GBP 45 624 using PVCE. Average total QALYs were 15.9 years for colonoscopy ± MRE and 16.3 years for PVCE.

## Discussion

For Crohn’s disease, highly effective treatment options are available but are associated with substantial costs, and for some patients duration of use may be limited [25]. Treat-to-target offers the opportunity to deliver improved long-term outcomes with mucosal healing generally accepted as that treatment target [2, 4, 26]. A commonly measured surrogate marker of mucosal healing is faecal calprotectin [11]. Its utility was recently demonstrated in the CALM study, which showed that disease management based on tight control of biomarkers and symptomatic assessment was associated with superior outcomes compared to symptomatic assessment alone [7].

In this context, we see capsule endoscopy as addressing an unmet need within Crohn’s disease care and advancing monitoring capabilities as it enables the physician to visualize the small bowel, which has been associated with disease activity in 11% of patients [3]. Small bowel capsule endoscopy is recommended by the European Crohn’s and Colitis Organisation (ECCO) for patients with suspected CD or increased faecal calprotectin levels and negative endoscopy [27]. PVCE has the potential to assess both the small and large bowel, making it an alternative to a combination of MRE and colonoscopy. Under current guideline recommendations, the use of both techniques should be based on clinicians’ discretion, and their relative advantages and disadvantages should be considered [28, 29]. In a publication by Greener *et al*. assessing the performance of MRE and capsule endoscopy on the reclassification of the disease capsule, endoscopy detected lesions in 51% of patients, while MRE detected lesions in only 25% of patients [30]. Kopylov *et al.* found that capsule endoscopy led to recommendations for changes in management in 52.3% of patients [31]. These recommendations included treatment onset or intensification in 82.5% of the patients [31], a finding that seems to be in agreement with the outcomes of our care pathway model.

As PVCE shows a higher sensitivity for inflammatory activity in the small bowel, potential flares in this region may be detected earlier and treated before a surgical intervention is necessary. The use of PVCE results in fewer surgeries in the model; the reduction in surgeries is driven by fewer colonic resections and emergency interventions but includes more early interventions such as abscess drainage and fistula repair. The timeliness of interventions is critical to patient well-being as a reduction in complications and a milder disease course may be associated with early treatment uptake [32]. Indeed, this model suggests a similar finding, as treatment costs align after the first few years, while surgery costs continue to rise with colonoscopy over time (Fig. 2). The flipside is that the use of PVCE may lead to increases in the cost of biologics. The proportion of patients using biologics at some point during the 20-year simulation was equivalent between the two monitoring modalities, but treatment was initiated earlier with PVCE. Nevertheless, as the majority of patients are expected to see gains in quality of life with use of PVCE, though results for individual patients do vary, the population benefit may be considerable.

Given that the pathway model mostly includes only hospital costs, the estimated cost of care is lower than in some previous publications, which have reported between £3000 and £6500 per patient per year [1, 33]. Costs may also be lower than some previous estimates given the introduction of biosimilars and other generic treatments in the Crohn’s care pathway. Supporting model validity, it was found that the rates of bowel resection in the model correspond well to those published by Frøslie *et al*. [6], data that were not used to inform the model development.

### Study limitations

A computational model is an aid to assess the consequences of the adaptation of a new treatment modality over a time horizon not feasible for a real-world trial. For certain parameters, insufficient data were available to inform the model and assumptions were required. Where made, the aim was to make these realistic but also conservative. There is also still debate around capsule retention and its impact, and no randomized, controlled trial data are available to inform the model. Therefore, as a proxy, a high and a low capsule retention rate reported in the literature was considered within this work, and multiple scenarios considering the use of the patency capsule and response to capsule retention were included. Still, an algorithm is not able to replace physicians’ judgement as to whether a patient is eligible for PVCE.

The diagnostic yield of PVCE, with its high sensitivity, is mainly derived from studies where it has been used in initial diagnosis [34, 35]. The predictive relationship of lesions identified by PVCE during monitoring is less well established, but a recent 2019 publication indicated that the endoscopic Lewis score may be a predictor for relapse and emergency hospitalization [36]. Nevertheless, given the relationship between mucosal healing and long-term outcome when assessed at colonoscopy, it seems reasonable to assume a similar relationship in this model. This only highlights, though, that more robust sensitivity and specificity data for the use of PVCE in Crohn’s disease monitoring are still required. This is especially true for colonic disease, where a recent review found that only five studies are reporting on capsule endoscopy in this setting and, of these, only two reported on sensitivity and specificity [37]. The first study used only the result of investigations in five patients, and the second was a paediatric population [38, 39]. Both studies had contradictory findings, as the first reported 40% and the second 100% specificity [38, 39]. In our work, no data specific to colonic identification were used. Instead, values from small bowel and combined small bowel and colonic studies were included, with a lower sensitivity and specificity applied in the colon than for small bowel disease detection.

## Conclusion

For monitoring of patients with diagnosed Crohn’s disease, PVCE provides direct assessment of mucosal healing and is likely to be considered a cost-effective alternative to colonoscopy ± MRE. The use of the patency capsule as standard is supported by model outcomes.
